# Association of Employment Disruptions and Financial Hardship Among Individuals Diagnosed with Cancer in the United States: Findings from a Nationally Representative Study

**DOI:** 10.1158/2767-9764.CRC-23-0157

**Published:** 2023-09-12

**Authors:** Michael T. Halpern, Janet S. de Moor, Xuesong Han, Jingxuan Zhao, Zhiyuan Zheng, K. Robin Yabroff

**Affiliations:** 1 Healthcare Delivery Research Program, NCI, Rockville, Maryland.; 2 Surveillance and Health Equity Science, American Cancer Society, Atlanta, Georgia.

## Abstract

Financial hardship (FH), defined as adverse patient effects due to cancer costs, is experienced by approximately half of individuals diagnosed with cancer. Many individuals diagnosed with cancer also experience disruptions with their employment. This study examines associations of employment disruptions and FH among a nationally representative sample of individuals diagnosed with cancer in the United States. We utilized 2016/2017 Medical Expenditure Panel Survey Experiences with Cancer data from individuals who worked for pay following cancer diagnosis. Employment disruption included taking extended paid time off work; switching to part-time/less demanding jobs; and/or retiring early due to cancer diagnosis/treatment. FH domains included: material (e.g., borrowing money/financial sacrifices); psychologic (e.g., worrying about medical bills/income); and behavioral (delaying/forgoing healthcare services because of cost). Multivariable logistic regression analyses determined associations of employment disruption and FH. Among 732 individuals with a cancer history, 47.4% experienced employment disruptions; 55.9% experienced any FH. Any FH was significantly more common among individuals with versus without employment disruptions across multiple measures and domains (68.7% vs. 44.5%; *P* value of difference <0.0001). Individuals with employment disruptions were more likely to have any FH [OR, 2.38; 95% confidence interval (CI), 1.62–3.52] and more FHs (OR, 2.76; 95% CI, 1.96–3.89]. This study highlights that employment disruptions are common and significantly associated with multiple domains of FH among individuals with a cancer history. Employer workplace accommodation, physician discussions regarding potential impacts of cancer care on employment, and other policies to minimize employment disruptions among individuals diagnosed with cancer may reduce FH in this vulnerable population.

**Significance:**

Individuals diagnosed with cancer may have employment disruptions; they may also develop FHs. People with cancer who have employment changes are more likely to also have FHs. Physicians and employers can help individuals with cancer through advancing planning, workplace assistance, and improved medical leave and insurance policies.

## Introduction

Advances in novel cancer treatments have accelerated growth in cancer care spending (1–4), including patient out-of-pocket spending (3, 5, 6), during the past decade in the United States. As a result, medical financial hardship, including difficulty paying medical bills, financial distress, and delayed or forgone medical care and prescription medications because of cost, is well documented among cancer survivors (7–15). Cancer treatment and lasting effects of treatment can also interfere with employment (16–18). Cancer survivors are more likely to report work limitations and missed work days than their peers without a cancer history, even many years following diagnosis (19–22). Approximately 41.3% of cancer survivors report requiring extended time away from work, facing challenges in performing usual duties, switching from full-time work to part-time work, or retiring from the work force earlier than expected (16). These employment disruptions can lead to loss of income as well as loss of access to employer-sponsored health insurance coverage (23), both of which are critical for ensuring that patients can afford their cancer treatments.

Several studies have reported that employment disruptions are associated with financial hardship among patients being treated for cancer and cancer survivors (18, 24). These studies were conducted in single states or within geographically-defined health systems (25) frequently among selected populations (25–27), such as adolescents and young adult cancer survivors or women with breast cancer. In addition, these studies frequently lacked comprehensive measurement of financial hardship, which precludes a nuanced understanding of the intersection between employment disruption and different aspects of financial hardship (24, 25, 27). One previous study used more detailed measures of financial hardship to evaluate associations with employment disruption (18) but did not restrict the study population to individuals working for pay at the time of or after cancer diagnosis, limiting the interpretation of results of this association. The current study complements and extends this earlier work. In this study, we used data from a nationally representative sample of individuals with a cancer diagnosis who worked for pay following their diagnosis to examine the associations between employment disruptions and multiple domains of financial difficulty, including material hardship and the psychologic and behavioral responses to that hardship.

## Materials and Methods

### Data Sources and Study Population

This study used data from the 2016 and 2017 Medical Expenditure Panel Survey (MEPS) Experiences with Cancer Survivorship Supplement (ECSS). MEPS is a nationally representative population-based survey collecting data on clinical and sociodemographic characteristics, health conditions, use of medical services, health insurance, income, and employment. Individuals completing the MEPS Household Component survey who had ever been told by a health professional that they had cancer/malignancy of any kind at age ≥18 years were asked to complete the ECSS. The sample design of the MEPS includes stratification, clustering, multiple stages of selection, and oversampling. Furthermore, the MEPS sampling weights reflect adjustments for survey nonresponse and adjustments to population control totals. Information about computing SEs for MEPS estimates based on the complex sample design is available as https://meps.ahrq.gov/survey_comp/standard_errors.jsp. Additional information on the MEPS ECSS, including links to survey instruments and sites for downloads of data and analysis weights, is available at https://healthcaredelivery.cancer.gov/meps/. As the MEPS data are available for public download without any charge and do not contain any Protected Health Information or Personally Identifiable Information, these data do not meet criteria for human subjects research and no Institutional Review Board review was required.

As the study focused on employment disruptions (discussed below), only ECSS respondents who responded “Yes” to the question “At any time from when you were first diagnosed with cancer until now, were you working for pay at a job or business?” were included in the study sample. ECSS respondents with non-melanoma or unknown-type skin cancer or who indicated that they were never treated for cancer (and thus may have been incorrectly categorized as having cancer) were excluded.

### Study Outcome Measure

The study outcome measure was financial hardship, based on responses to ECSS questions about the effects of the cancer diagnosis or treatment: (i) material financial hardship, including having to borrow money or go into debt, making financial sacrifices (i.e., reducing spending on basics such as food or clothing, using savings set aside for other purposes, or making changes to living situation), or being unable to cover the patient's share of the costs of medical care, (ii) psychologic financial hardship, including worrying about paying large medical bills, worrying about family's financial stability, or concern about keeping job and income or limitations of future earnings; and (iii) behavioral financial hardship, including delaying, forgoing, or making other changes because of cost to cancer prescription medicine, specialist visits, treatment, follow-up care, mental health services, or other care.

Each financial hardship domain—material, psychologic, and behavioral—was evaluated separately. The main study outcome measures were any financial hardship (i.e., any of the three domains) and number of financial hardships (count of the different domains of financial hardships experienced, categorized as zero, one, or two or more).

### Exposure Variable

The primary exposure variable was employment disruption. This was defined by ECSS questions indicating respondents had, because of their cancer, cancer treatment, or cancer lasting effects, taken extended paid time off work; switched to a part-time or less demanding job or to a flexible work schedule; and/or retired earlier than planned.

### Study Population Characteristics

Other respondent characteristics included sex (male/female), race/ethnicity (non-Hispanic White vs. all other races/ethnicities), age group (18–39, 40–54, 55–64, 65 or older), educational attainment (less than high school, high school graduate, any college education), current marital status (married/not married), number of MEPS priority health conditions (select medical conditions specified by the Agency for HealthCare Research and Quality for their prevalence, expense, or relevance to policy, coded in this study as zero, one or two or more priority conditions excluding cancer), time since last cancer treatment [<1 year ago (including current treatment), 1 to <5 years, 5 or more years ago, or time since last treatment missing], and cancer site (breast, cervical, colon, melanoma, prostate, uterus, or other, with other including all cancer types reported by less than 5% of the study population). Health insurance at cancer diagnosis is also included as a respondent characteristic, coded as the following mutually exclusive categories: any private health insurance (with or without other types of insurance); Medicare without any private insurance; Medicaid or other public insurance coverage without private insurance or Medicare coverage; or uninsured. In addition, as the population with Medicare coverage prior to age 65 (eligible due to permanent disability or certain chronic conditions) is very different than the population with Medicare coverage at age 65 years or older (age eligible for coverage), we have included separate covariates for Medicare coverage at cancer diagnosis with estimated age of diagnosis before 65 versus 65 or older. Because the MEPS and the ECSS do not include age at cancer diagnosis, age at diagnosis was estimated using age at survey completion and estimated years since last cancer treatment. Years since last cancer treatment is included as a categorical variable in the ECSS; estimated years since cancer diagnosis based on the categorical responses to this variable are presented in Supplementary Table S1.

### Study Analyses

We performed descriptive analyses using Rao-Scott *χ*^2^ tests to compare financial hardship measures by employment disruption status and used multivariable logistic regression analyses to examine associations of any financial hardship and number of financial hardships (0/1/2+) with employment disruption and patient characteristics. Patient characteristics included in regressions were sex, race/ethnicity, age groups, education, current marital status, number of MEPS priority medical conditions, time since last cancer treatment, insurance at cancer diagnosis, and cancer type. For cancer type, melanoma was used as the reference category in regression analyses as the cancer type occurring among both men and women reported by the largest proportion of the study population.

Two secondary multivariable analyses were performed. First, as impacts of employment disruption on financial hardship may differ between men and women, we examined differences in associations between employment disruptions and hardship stratified by sex. Second, as the effects on financial hardship may vary based on time since employment disruption, we performed subgroup analysis for individuals with <1 year versus ≥1 year following last cancer treatment.

All analyses used sample weights provided with the ECSS to provide nationally representative estimates and address survey nonresponse. Analyses were performed using PROC SURVEYFREQ and SURVEYLOGISTIC in SAS 9.4, with adjustment for the complex survey design of the MEPS.

### Data Availability

The data analyzed in this study were obtained from the MEPS website; links to the MEPS ECSS data are at https://healthcaredelivery.cancer.gov/meps/.

## Results

### Study Population Characteristics and Employment Disruption

Table 1 presents characteristics of the 732 individuals in the study population. The largest age group was among individuals aged 65 years and older; 58.5% were female. A majority were non-Hispanic White, currently married, and had had at least some college education. More than half (57%) were currently employed and approximately two-thirds had private insurance (with or without other types of insurance) at the time of their cancer diagnosis. Most had two or more other medical conditions and had completed treatment at least 5 years prior to the survey. Supplementary Table S2 presents the proportion of individuals with employment disruption and financial hardship by sociodemographic and clinical characteristics.

**TABLE 1:** Characteristics of the study population

	*N*	Weighted %
Total	732	100
Age group		
18–39	67	7.9
40–54	149	21.2
55–64	210	29.7
≥65	306	41.2
Female	437	58.5
Race/ethnicity		
Non-Hispanic White only	523	80.0
All other race/ethnicities	209	20.0
Current marital status		
Married	422	61.6
Not married^a^	310	38.4
Education		
Less than high school graduate	66	6.3
High school graduate	192	23.7
Some college or more	474	70.0
Current employment		
In labor force	390	57.1
Not in the labor force	342	42.9
Health insurance at cancer diagnosis		
Any private	493	69.8
Medicare, no private, age at diagnosis <65	20	2.0
Medicare, no private, age at diagnosis 65+	30	4.3
Medicaid and other non-Medicare public only	77	10.0
Uninsured	110	13.7
Number of known MEPS priority conditions (excluding cancer)^b^		
0	64	9.7
1	118	15.6
2+	550	74.7
Years since last cancer treatment		
<1 (including currently treated)	154	20.8
1 to <5	131	17.3
≥5	398	55.5
Missing	49	6.5
Cancer site		
Breast	210	29.4
Cervical	61	8.1
Colon	52	6.9
Melanoma	65	9.8
Prostate	117	15.6
Uterus	42	4.9
All other sites (each <5% of total population)	217	29.6

^a^Not married included widowed, divorced, separated, or never married.

^b^Conditions include arthritis, asthma, diabetes, emphysema, heart disease (angina, coronary heart disease, heart attack, and other heart condition/disease), high cholesterol, hypertension, and stroke.

Table 2 provides details regarding employment disruptions. More than one-third of the study population reported taking extended paid time off due to their cancer, cancer treatment, or cancer lasting effects. Approximately 16% indicated having switched to a part-time or less demanding job or to a flexible work schedule and 27% to having retired early. Overall, 47.4% reported at least one employment disruption due to cancer based on these measures.

**TABLE 2:** Types of employment disruption reported by study population

Type of disruption	Number	Weighted %
Took extended paid time off	253	35.6
Switched to part time, change to less demanding job, or to a flexible work schedule	111	15.9
Retired earlier than planned	187^a^	27.2
Reported any of the above types of employment disruptions	356	47.4

^a^Missing = 114.

### Association of Employment Disruptions and Financial Hardship

Table 3 presents each domain of financial hardship and the components of each financial hardship domain, overall and by the presence and absence of employment disruption, and the unadjusted association of financial hardship and employment disruption. Material financial hardship was reported by 34.9% of the study population, with the largest contributing factor being making financial sacrifices. Psychologic financial hardship was reported by 40.0% and behavioral financial hardship by 26.9%. Overall, 55.9% of the study population reported any financial hardship: 23.5% experienced one category of financial hardship while 32.4% experienced two or three categories of financial hardship.

**TABLE 3:** Differences in financial hardship by employment disruption^a^

	*N* (unweighted)	% (95% CI)	Employment disruptions % (95% CI)	No employment disruptions % (95% CI)	*P*
Material financial hardship					
Had to borrow money or go into debt	78	9.7 (7.6–11.8)	17.2 (14.8–19.6)	3.0 (0.5–2.0)	<0.0001
Made financial sacrifices	402	29.2 (25.7–32.7)	45.6 (41.3–49.9)	14.4 (11.5–17.3)	<0.0001
Unable to cover share of the costs of medical care	105	12.7 (10.0–15.4)	18.9 (14.7–23.0)	7.1 (4.8–9.4)	<0.0001
Any material financial hardship	420	34.9 (30.9–38.8)	51.7 (47.0–56.5)	19.7 (16.0–23.3)	<0.0001
Psychologic financial hardship					
Worried about paying large medical bills	234	29.5 (25.9–33.1)	41.8 (37.0–46.5)	18.3 (15.5–21.1)	<0.0001
Worried about family's financial stability	226	30.3 (26.9–33.7)	44.2 (39.6–48.8)	17.8 (15.2–20.3)	<0.0001
Concerned about keeping job and income or earnings	198	26.3 (23.1–29.5)	40.1 (35.5–44.8)	13.7 (11.3–16.0)	<0.0001
Any psychologic financial hardship	302	40.0 (36.3–43.7)	55.6 (50.3–60.8)	25.9 (22.7–29.1)	<0.0001
Behavioral financial hardship					
Delay/forgo/make other changes to the following cancer care because of cost					
Prescription medicine	56	6.3 (4.4–8.2)	8.6 (7.0–10.2)	4.3 (2.7–5.9)	0.0357
Visit to specialist	57	7.0 (5.0–9.0)	9.1 (7.3–11.0)	5.1 (3.9–6.3)	0.0453
Treatment (other than prescription medicine)	27	3.2 (2.1–4.3)	5.7 (4.3–7.1)	0.9 (0.5–1.4)	<0.0001
Follow-up care	77	8.6 (6.8–10.4)	9.7 (7.9–11.5)	7.6 (5.4–9.8)	0.3164
Mental health services	25	2.7 (1.7–3.6)	4.8 (3.9–5.7)	0.7 (0.6–0.8)	<0.0001
Other	79	9.3 (7.0–11.5)	8.0 (6.2–9.7)	10.4 (7.3–13.5)	0.2604
Any behavioral hardship	225	26.9 (23.4–30.4)	29.6 (26.7–32.4)	24.5 (20.5–28.5)	0.1440
Any financial hardship	430	55.9 (52.0–59.9)	68.7 (64.1–73.2)	44.5 (40.2–48.8)	<0.0001
One domain of financial hardship	176	23.5 (19.9–27.1)	21.3 (17.5–25.0)	25.5 (21.9–29.2)	0.1620
Two or three domains of financial hardship	254	32.4 (28.9–36.0)	47.4 (42.7–52.2)	19.0 (15.5–22.4)	<0.0001

^a^Employment disruptions measured as taking extended paid time off work; switched to a part-time or less demanding job or to a flexible work schedule; and/or retired earlier than planned because of their cancer, cancer treatment, or cancer lasting effects.

All components of material and psychologic financial hardship domains were more prevalent among those with employment disruptions (all *P* < 0.0001). Overall, the prevalence of any financial hardship was significantly greater among those with employment disruptions than those without employment disruptions (68.7% vs. 44.5%, respectively, *P* < 0.0001). Almost half of individuals with employment disruptions experienced two or more financial hardships (47.4%), while fewer than one-fifth (19.0%) of those without employment disruptions reported two or more financial hardships (*P* < 0.0001).

### Multivariable Analyses of Association of Employment Disruptions and Financial Hardship

Table 4 presents results from multivariable logistic regression analyses of associations of any financial hardships with employment disruption following cancer diagnosis, controlling for sociodemographic and clinical characteristics. Among individuals who experienced employment disruptions, the odds of experiencing any financial hardships is 2.38 greater than that for individuals without employment disruptions (*P* < 0.0001). Individuals with race/ethnicity other than non-Hispanic White (vs. non-Hispanic Whites) and with a history of colon cancer, prostate cancer, or cancer of sites in the “other group” (vs. those with melanoma) also had significantly increased odds of experiencing any financial hardship. Survivors in the 65+ age group had significantly decreased odds of experiencing any financial hardship compared with survivors ages 18–39. Sex, marital status, education, health insurance at diagnosis, number of other conditions, and years since last treatment were not significantly associated with reporting any financial hardship. Multivariable logistic regression analyses examining the association of employment disruption with each of the three financial hardship domains (material, psychologic, and behavioral) that comprise “any financial hardship” are presented in Supplementary Tables S3–S5.

**TABLE 4:** Multivariable logistic model examining associations with any financial hardships (OR, 95% CI)^a^

	Odds of any financial hardships	
	OR	95% CI
Employment disruption		
Disruption	2.38	1.62, 3.52
No disruption (reference)	—	—
Age group		
18–39 (reference)	—	—
40–54	1.07	0.50–2.3
55–64	0.68	0.33–1.42
≥65	0.37	0.19–0.74
Sex		
Male (reference)	—	—
Female	1.36	0.79–2.33
Race/ethnicity		
Non-Hispanic white only (reference)	—	—
All other race/ethnicities	2.18	1.36–3.49
Current marital status		
Married	0.78	0.53–1.14
Not married (reference)	—	—
Education		
Less than high school graduate (reference)	—	—
High school graduate	0.89	0.30–2.63
Some college or more	0.62	0.23–1.71
Health insurance at cancer diagnosis		
Any private (reference)	—	—
Medicare, no private, age at diagnosis <65	1.99	0.79–5.06
Medicare, no private, age at diagnosis 65+	2.03	0.85–4.83
Medicaid and other non-Medicare public only	0.82	0.39–1.72
Uninsured	1.05	0.58–1.9
Number of known MEPS priority conditions (excluding cancer)^b^		
0 (reference)	—	—
1	1.07	0.53–2.15
2+	1.82	0.95–3.5
Years since last cancer treatment		
<1 (reference)	—	—
1 to <5	1.20	0.65–2.25
≥5	0.81	0.49–1.34
Missing	0.90	0.38–2.13
Cancer site		
Melanoma (reference)	—	—
Breast	1.42	0.68–2.95
Cervical	1.54	0.61–3.84
Colon	3.28	1.29–8.34
Prostate	2.75	1.30-5.83
Uterus	0.63	0.28–1.41
All other sites	4.36	2.14–8.88

^a^Results from multivariable logistic model controlling for sex, race/ethnicity, age group, educational attainment, current marital status, health insurance at the time of cancer diagnosis, number of MEPS priority health conditions, time since last cancer, cancer site, and employment disruption status.

^b^Conditions include arthritis, asthma, diabetes, emphysema, heart disease (angina, coronary heart disease, heart attack, and other heart condition/disease), high cholesterol, hypertension, and stroke.

Table 5 presents results from multivariable ordinal logistic regression analyses of associations of number of financial hardships (categorized as zero, one, or two or more) with employment disruption following cancer diagnosis, controlling for sociodemographic and clinical characteristics. Among individuals who experienced employment disruptions, the odds of experiencing more financial hardships is 2.76 greater than that for those without employment disruptions (*P* < 0.0001). Similar to analyses of any financial hardship, individuals from racial/ethnic populations other than non-Hispanic White and with a history of colon cancer, prostate cancer, or cancer of sites in the “other group” (vs. those with melanoma) also had significantly increased odds of more financial hardships, while those over age 65 years (compared with those age 18–39 years) had significantly decreased odds of more financial hardships. However, in contrast to findings from the analysis of any financial hardship, individuals who had Medicare coverage when diagnosed with cancer at age <65 or a history of breast cancer also had significantly increased odds of more financial hardships while those who were currently married had significantly lower odds of more financial hardships.

**TABLE 5:** Multivariable logistic model examining associations with number of financial hardships (OR, 95% CI)^a^

	Odds of more financial hardships	
	OR	95% CI
Employment disruption		
Disruption	2.76	1.96–3.89
No disruption (reference)	—	—
Age group		
18–39 (reference)	—	—
40–54	0.84	0.44–1.58
55–64	0.66	0.35–1.22
≥65	0.34	0.19–0.60
Sex		
Male (reference)	—	—
Female	1.07	0.69–1.67
Race/ethnicity		
Non-Hispanic White only (reference)	—	—
All other race/ethnicities	1.89	1.22–2.93
Current marital status		
Married	0.67	0.48–0.93
Not married (reference)	—	—
Education		
Less than high school graduate (reference)	—	—
High school graduate	0.73	0.31–1.72
Some college or more	0.59	0.26–1.33
Health insurance at cancer diagnosis		
Any private (reference)	—	—
Medicare, no private, age at diagnosis <65	3.37	1.51–7.53
Medicare, no private, age at diagnosis 65+	1.91	0.84–4.33
Medicaid and other non-Medicare public only	0.92	0.50–1.67
Uninsured	1.26	0.77–2.06
Number of known MEPS priority conditions (excluding cancer)^b^		
0 (reference)	—	—
1	0.88	0.48–1.65
2+	1.33	0.77–2.32
Years since last cancer treatment		
<1 (reference)	—	—
1 to <5	1.34	0.8–2.22
≥5	0.94	0.64–1.40
Missing	1.10	0.52–2.32
Cancer site		
Melanoma (reference)	—	—
Breast	2.02	1.03–3.95
Cervical	2.22	0.94–5.24
Colon	3.96	1.77–8.89
Prostate	2.88	1.49–5.57
Uterus	0.86	0.4–1.83
All other sites	5.19	2.76–9.76

^a^Results from multivariable ordinal logistic model controlling for sex, race/ethnicity, age group, educational attainment, current marital status, health insurance at the time of cancer diagnosis, number of MEPS priority health conditions, time since last cancer, cancer site, and employment disruption status.

^b^Conditions include arthritis, asthma, diabetes, emphysema, heart disease (angina, coronary heart disease, heart attack, and other heart condition/disease), high cholesterol, hypertension, and stroke.

### Secondary Analyses

Information on the timing of employment disruptions following cancer diagnosis is not available in the MEPS ECSS. While years following last cancer treatment were not significantly associated with number of financial hardships in multivariable regression analyses (Table 4), we examined the association between employment disruptions and either any financial hardship or number of financial hardships separately for survivors within 1 year of last treatment versus those with longer times since last treatment. In regression analyses, employment disruption remained significantly associated with any financial hardship among survivors with ≥1 year following last treatment (OR, 2.59; *P* < 0.0001). However, in separate regression analyses of survivors with <1 year following last treatment, the odds for any financial hardship with employment disruption was marginally not statistically significant (OR, 1.79; *P* = 0.10). In similar multivariable regression analyses examining associations of time since last treatment with number of financial hardships, both those with <1 year and those with ≥1 years since last treatment who experienced employment disruption had significantly increased odds of more financial hardships (OR, 3.48; *P* = 0.0002 and OR, 2.68; *P* < 0.0001, respectively).

Impacts of employment disruption on financial hardship may differ between men and women. Although sex was not significantly associated with either of the financial hardship measures (Tables 4 and 5), we also conducted analyses to examine interactions between sex and employment disruption on associations with financial hardship. In multivariable regression analyses, the interaction term between sex and employment disruption was not significantly associated with odds of financial hardship (*P* = 0.81) or odds of more financial hardships (*P* = 0.82).

## Discussion

In this nationally representative study, we found that employment disruptions, including extended time off from work, switch to part-time work, and early retirement, were associated with financial hardship among individuals with a cancer history in the United States. Our findings were consistent across multiple measures and domains of financial hardship. Findings of the association between employment disruption and financial hardship were also consistent across survivors’ characteristics that could influence development of financial hardship: a sex by employment disruption interaction term was not significantly associated with financial hardship and analyses stratified by time since last treatment showed only a marginal difference in associations with one of the two financial hardship outcomes. Nearly half of the sample who worked at any time since cancer diagnosis experienced any type of employment disruption due to cancer. With a growing population of cancer survivors (28) and increasing patient out-of-pocket burden associated with cancer treatments (3, 5, 6, 29), prevalence of financial hardship will likely increase in the future. Addressing and mitigating financial hardship is especially important because accumulating evidence suggests that medical financial hardship is associated with poor health outcomes, including worse quality of life (30) and survival following cancer diagnosis (31, 32). Many efforts to mitigate financial hardship are conducted within the health care setting and address patient out-of-pocket costs; our findings suggest that interventions and policies to help workers maintain employment may also help to mitigate financial hardship among individuals diagnosed with cancer.

Workplace policies can play an important role in access to health care and in mitigating the impact of cancer on employee's ability to work. Employers make decisions about whether to offer health insurance coverage and paid sick leave to their workers in the United States as well as decisions related to retirement benefits for workers. Among women who were employed at the time of diagnosis of breast cancer, those with no sick leave were more than three times as likely to lose their employment as were those with paid sick leave (33). In addition, employers can choose to offer workplace accommodations to their workforce, such as flexible hours, flexible locations, and changes in responsibilities and duties (34, 35). Results from a large U.K.-based survey indicate that individuals with flexible work arrangements were more likely to continue working during cancer treatment and those who had a return to work meeting with their employer were more likely to return to work following treatment (36). Previous research has indicated that individuals diagnosed with cancer who want to return to work following treatment experience many barriers; these barriers are mainly cancer or treatment related in the time period close to diagnosis, but years later, these barriers tend to be work related (37). Provision of additional workplace supports and collaboration between employers and individuals diagnosed with cancer may facilitate return to work (38).

Employers should also actively counter discrimination experienced by cancer survivors in the workplace. Perceived employer discrimination is associated with decreased rates of return to work (39), and cancer survivor may be more likely to perceive they are being stigmatized in the workplace than do health care/vocational service providers or employers (40). Previous research has documented that individuals with cancer, compared with those with other disabilities, were more likely to allege discrimination in unlawful discharge, demotion, wages, layoff, benefits, and referrals; individuals with cancer were also significantly more likely to have Equal Employment Opportunity Commission investigations find that discrimination had occurred (41). Younger (vs. older) individuals with cancer were more likely to allege discrimination in promotion, training, reinstatement, and referrals and less likely to allege discrimination in benefits (42). These findings suggest that workplace discrimination against cancer survivors occurs, may enhance the likelihood of employment disruption among survivors, and could increase the likelihood of financial hardship.

Workplace accommodations are associated with return to work following completion of cancer treatment in patients (43). Currently, many employed cancer survivors lack workplace accommodations and more than one-third lack paid sick leave; survivors with lower household income, without health insurance, working part-time or in small businesses (<50 employees) are most likely to lack paid sick leave (44). The federal Family and Medical Leave Act requires that employers with more than 50 employees provide certain workers with up to 12 weeks of unpaid leave (https://www.dol.gov/agencies/whd/fmla). Unpaid leave can help people maintain employment and health insurance coverage, but it is likely less helpful in mitigating financial hardship than paid leave. Some states require employers to offer more generous unpaid leave (45). In addition, multiple states, cities, and counties have laws guaranteeing paid sick leave (46) and a small number of states mandate disability insurance requirements (47). To date, little is known about the effects of federal, state, and local leave and disability insurance policies for employment and financial hardship among cancer survivors and informal caregivers; this will be an important area for future research.

Our findings also have implications for oncology care providers and consideration of patient employment and work duties when discussing expected benefits and risks of different treatment options to their patients. National Comprehensive Cancer Network treatment guidelines recommend discussing the potential impact of cancer care on employment (48). These discussions will be particularly relevant when treatment adjustments can help patients minimize time away from work and maintain employment, especially if they do not have paid sick leave. An individual's employment may also be part of their identity; mitigating disruption to employment may have a range of impacts on a cancer patient's mental health, quality of life, and well-being (49, 50). Previous studies suggest that almost two-thirds of cancer survivors who were working at the time of diagnosis discussed employment with any health care provider since diagnosis (51). Estimates of more extensive employment communication are far lower (52). Receiving advice from one's doctor about work is associated with returning to work following treatment (36). Thus, research is needed to better incorporate employment conversations into cancer care and to inform the content and frequency of those conversations.

Findings reported here are consistent with those from a previous study (18) that used only a subset of the MEPS data included in the current study. This previous study limited the study sample examined for employment disruptions to individuals ages 18–64 and did not restrict analyses to those who were employed at the time of or following cancer diagnosis. The current study, by expanding the sample to all adults diagnosed with cancer, limiting analyses to individuals employed at cancer diagnosis, and including a larger group of cancer survivors, provides robust information on employment disruptions and financial hardship generalizable to all U.S. cancer survivors who worked for pay at the time of or after diagnosis.

Our findings of inverse associations between socioeconomic status and financial hardship are consistent with previous research (18, 24). People with more education are more likely to work in white-collar positions and for employers who offer paid sick leave and health insurance benefits (53, 54), which may be protective against financial hardship. We also found that people who are individuals from racial/ethnic minority populations were more likely to report financial hardship than their non-Hispanic White counterparts. Other research has shown that non-Hispanic Black and Hispanic individuals diagnosed with cancer were more likely to report employment-related income loss than were non-Hispanic White individuals (55). Future research examining associations of employment benefits and workplace accommodations and access to and receipt of cancer care among working age adults is warranted.

Consistent with many previous studies, our analyses did not find significant associations between time since last treatment and financial hardship (Tables 4 and 5). However, the moderating effect of time since last treatment on the relationship between employment disruption and financial hardship was inconsistent: time since last treatment did not have a moderating effect on the relationship between employment disruption and number of financial hardships but did on the relationship between employment disruption and any financial hardships. That is, in analysis of the association between employment disruption and any financial hardships, a significant relationship was found only among survivors with >1 year following last treatment. Although interesting, these results should be interpreted cautiously and confirmed in other studies; the subgroup of survivors who were treated within the previous year and experienced employment disruption was small, and the association between employment disruption and financial hardship among this subgroup was only marginally nonsignificant (*P* = 0.10). Few other studies have examined the potential differentiating effects of time on variables associated with financial hardship. For example, Hastert and colleagues (56) reported that health-related quality of life (HRQOL) was lower among individuals experiencing financial hardship for survivors diagnosed within 18 months, but no association between HRQOL and financial hardship was observed for survivors diagnosed more than 18 months prior. More research is needed to fully examine how effects of employment disruptions over time and timing of employment disruptions may impact financial hardship.

This study has limitations. The MEPS ECSS survey is cross-sectional and no information is available about temporality; we cannot infer causality about the associations between employment disruptions and financial hardship. The survey did not contain information about cancer stage at diagnosis or treatment(s); those data are not included in the MEPS ECSS. Individuals with more advanced stage disease at diagnosis and those receiving more intense treatment may be more likely to experience employment disruption. However, the current study did control for time since last treatment in multivariable regression analyses. Individuals with more advanced stage at diagnosis have reduced life expectancy, yet more than half the study population reported at least 5 years since last treatment (Table 1), suggesting that a majority of the study population were not diagnosed with advanced stage disease.

MEPS also does not contain information about prediagnosis employment status or economic position, which may be related to both employment disruptions and financial hardship. No information is available in the MEPS regarding timing of employment disruptions other than occurrence following cancer diagnosis. Measures are self-reported and subject to recall bias. Self-reported measures may be best for some aspects of financial hardship (e.g., worry about paying large medical bills) which are not available from other sources, however. Given the limitations of the MEPS ECSS, future research including detailed information about stage of diagnosis, type and timing of treatment(s), and employment and financial hardship in prospective cohorts may help to disentangle complex relationships with treatment-related impairments and economic and health outcomes among cancer survivors.

Despite these limitations, this study has important implications for improving care and mitigating financial hardship for working patients with a cancer diagnosis and cancer survivors. Our findings suggest that interventions and policies to help workers maintain employment, including paid sick leave and workplace accommodations, are needed. Furthermore, oncology care providers should expand discussions of cancer treatment risks and benefits to include patient ability to maintain employment during informed patient-physician decision-making.

## Supplementary Material

Supplementary Table 1 Supplementary Table 1

Supplementary Table 2 Supplementary Table 2

Supplementary Table 3 Supplementary Table 3

Supplementary Table 4 Supplementary Table 4

Supplementary Table 5 Supplementary Table 5
