# Supplementary material for: Association of Employment Disruptions and Financial Hardship Among Individuals Diagnosed with Cancer in the United States: Findings from a Nationally Representative Study
Source: Cancer Res Commun. 2023 Sep 12;3(9):1830–9. doi: 10.1158/2767-9764.CRC-23-0157 (PMC10496757; doi:10.1158/2767-9764.CRC-23-0157)
Supplement: Supplementary Table 1 [file crc-23-0157-s01.docx]

**Supplementary Table 1: Years Since Last Cancer Treatment Response Options used to Estimate Years Since Cancer Diagnosis**

| **ECSS Response Options to Question "About how long ago did you receive your last cancer treatment?”** | **Estimated Years since Cancer Diagnosis** |
| --- | --- |
| Currently being treated | 0 |
| <1 | 0 |
| 1 to <3 | 2 |
| 3 to <5 | 4 |
| 5 to <10 | 8 |
| 10 to 20 | 15 |
| >20 | 25 |
