# Supplementary material for: Association of Employment Disruptions and Financial Hardship Among Individuals Diagnosed with Cancer in the United States: Findings from a Nationally Representative Study
Source: Cancer Res Commun. 2023 Sep 12;3(9):1830–9. doi: 10.1158/2767-9764.CRC-23-0157 (PMC10496757; doi:10.1158/2767-9764.CRC-23-0157)
Supplement: Supplementary Table 2 [file crc-23-0157-s02.docx]

**Supplementary Table 2: Proportion of Study Population with Employment Disruption or Any Financial Hardship by Sociodemographic and Clinical Characteristics**

|  | **Unadjusted Weighted Proportions** | |
| --- | --- | --- |
|  | **% with Employment Disruption** | **% with any Financial Hardship** |
| **Age group** |  |  |
| 18-39 | 49.4% | 65.0% |
| 40-54 | 66.7% | 72.1% |
| 55-64 | 45.7% | 56.5% |
| ≥ 65 | 38.2% | 45.6% |
| **Sex** |  |  |
| Male | 43.1% | 57.4% |
| Female | 50.4% | 54.9% |
| **Race / ethnicity** |  |  |
| Non-Hispanic white only | 44.5% | 51.8% |
| All other race/ethnicities | 58.9% | 72.7% |
| **Current marital status** |  |  |
| Married | 49.9% | 54.1% |
| Not married | 43.2% | 58.9% |
| **Education** |  |  |
| Less than high school graduate | 35.7% | 65.6% |
| High school graduate | 47.7% | 62.1% |
| Some college or more | 48.3% | 53.0% |
| **Health insurance at cancer diagnosis** |  |  |
| Any private | 50.0% | 55.1% |
| Medicare with diagnosis age<65, no private | 58.0% | 81.0% |
| Medicare with diagnosis age 65+, no private | 26.2% | 56.1% |
| Medicaid and other non-Medicare public only | 48.6% | 56.7% |
| Uninsured | 38.8% | 56.4% |
| **Number of known MEPS priority conditions (excluding cancer)**† |  |  |
| 0 | 50.7% | 51.9% |
| 1 | 51.5% | 53.3% |
| 2+ | 46.1% | 57.0% |
| **Years since last cancer treatment** |  |  |
| <1 | 54.0% | 62.9% |
| 1 to <5 | 62.8% | 68.5% |
| ≥5 | 40.6% | 50.5% |
| Missing | 42.7% | 46.8% |
| **Cancer site** |  |  |
| Breast | 60.5% | 48.8% |
| Cervical | 18.3% | 47.9% |
| Colon | 54.7% | 65.3% |
| Melanoma | 13.6% | 34.5% |
| Prostate | 38.8% | 54.3% |
| Uterus | 59.7% | 38.1% |
| All other sites (each <5% of total population) | 59.9% | 73.2% |

† Conditions include arthritis, asthma, diabetes, emphysema, heart disease (angina, coronary heart disease, heart attack, and other heart condition/disease), high cholesterol, hypertension, and stroke.
