# Supplementary material for: Association of Employment Disruptions and Financial Hardship Among Individuals Diagnosed with Cancer in the United States: Findings from a Nationally Representative Study
Source: Cancer Res Commun. 2023 Sep 12;3(9):1830–9. doi: 10.1158/2767-9764.CRC-23-0157 (PMC10496757; doi:10.1158/2767-9764.CRC-23-0157)
Supplement: Supplementary Table 4 [file crc-23-0157-s04.docx]

**Supplementary Table 4**. **Multivariable Logistic Model Examining Associations with Psychological Financial Hardships (odds ratio, 95% confidence interval)** †

|  | **Odds of Psychological Financial Hardship** | |
| --- | --- | --- |
|  | **OR** | **95 % CI** |
| **Employment disruption** |  |  |
| Disruption | 2.89 | 1.95, 4.27 |
| No disruption (reference) | -------------------- | -------------------- |
| **Age group** |  |  |
| 18-39 (reference) | -------------------- | -------------------- |
| 40-54 | 1.07 | 0.52, 2.19 |
| 55-64 | 0.80 | 0.40, 1.58 |
| ≥ 65 | 0.28 | 0.14, 0.55 |
| **Sex** |  |  |
| Male (reference) | -------------------- | -------------------- |
| Female | 0.86 | 0.52, 1.45 |
| **Race / ethnicity** |  |  |
| Non-Hispanic white only (reference) | -------------------- | -------------------- |
| All other race/ethnicities | 1.93 | 1.16, 3.19 |
| **Current marital status** |  |  |
| Married | 0.62 | 0.41, 0.93 |
| Not married (reference) | -------------------- | -------------------- |
| **Education** |  |  |
| Less than high school graduate (reference) | -------------------- | -------------------- |
| High school graduate | 0.78 | 0.32, 1.92 |
| Some college or more | 0.84 | 0.34, 2.07 |
| **Health insurance at cancer diagnosis** |  |  |
| Any private (reference) | -------------------- | -------------------- |
| Medicare, no private, age at diagnosis <65 | 2.11 | 0.88, 5.07 |
| Medicare, no private, age at diagnosis 65+ | 1.36 | 0.41, 4.53 |
| Medicaid and other non-Medicare public only | 0.73 | 0.34, 1.57 |
| Uninsured | 1.22 | 0.71, 2.10 |
| **Number of known MEPS priority conditions (excluding cancer)**†† |  |  |
| 0 (reference) | -------------------- | -------------------- |
| 1 | 0.68 | 0.32, 1.47 |
| 2+ | 1.84 | 0.99, 3.44 |
| **Years since last cancer treatment** |  |  |
| <1 (reference) | -------------------- | -------------------- |
| 1 to <5 | 1.31 | 0.77, 2.24 |
| ≥5 | 0.72 | 0.46, 1.13 |
| Missing | 0.66 | 0.29, 1.50 |
| **Cancer Site** |  |  |
| Melanoma (reference) | -------------------- | -------------------- |
| Breast | 3.16 | 1.25, 7.98 |
| Cervical | 2.02 | 0.75, 5.40 |
| Colon | 6.42 | 2.33, 17.71 |
| Prostate | 3.21 | 1.36, 7.59 |
| Uterus | 0.98 | 0.40, 2.45 |
| All other sites | 5.53 | 2.3, 13.32 |

† Results from multivariable logistic model controlling for sex, race/ethnicity, age group, educational attainment, current marital status, health insurance at the time of cancer diagnosis, number of MEPS priority health conditions, time since last cancer, cancer site, and employment disruption status.

†† Conditions include arthritis, asthma, diabetes, emphysema, heart disease (angina, coronary heart disease, heart attack, and other heart condition/disease), high cholesterol, hypertension, and stroke.
